# TNFAIP3 gene rs10499194, rs13207033 polymorphisms decrease the risk of rheumatoid arthritis

**DOI:** 10.18632/oncotarget.12638

**Published:** 2016-10-13

**Authors:** Ming-Jie Wang, Hao-Yu Yang, Hui Zhang, Xindie Zhou, Rui-Ping Liu, Yuan-Yuan Mi

**Affiliations:** ^1^ Department of Orthopedics, Affiliated Hospital of Nanjing Medical University, Changzhou Second People's Hospital, Changzhou, China; ^2^ Department of Urology, The Third Affiliated Hospital of Nantong University, Wuxi, PR China

**Keywords:** TNFAIP3, rheumatoid arthritis, meta-analysis, single nucleotide polymorphism

## Abstract

Accumulating evidences suggested that tumor necrosis factor alpha inducible protein 3 (TNFAIP3) gene rs10499194, rs13207033 polymorphisms may be associated with the risk of rheumatoid arthritis (RA). However, these studies yielded contradictory findings. To clarify convincing associations, we conducted a comprehensive meta-analysis by searching in PubMed, Embase, and the China Knowledge Resource Integrated Database. Pooled odds ratios (ORs) and 95% confidence intervals (CIs) were calculated by using fixed-effect or random-effect models. A total of 13 case-control studies for rs10499194 polymorphism and 6 studies for rs13207033 polymorphism were included. Our data indicated that TNFAIP3 gene rs10499194, rs13207033 polymorphisms were associated with the decreased risk of RA. Stratification analyses of ethnicity indicated rs10499194, rs13207033 polymorphisms decreased the risk of RA among Caucasian populations, but not among Asian populations. In conclusion, this meta-analysis indicates that TNFAIP3 gene rs10499194, rs13207033 polymorphisms decrease the risk of RA, especially among Caucasian populations.

## INTRODUCTION

Rheumatoid arthritis (RA) is an autoimmune inflammatory disease, which is characterized by chronic destructive inflammation in synovial joints. To date, the etiology of RA is poorly understood, but it is believed that complex genetic and environmental factors play important roles in the pathogenesis of RA [1]. Previous studies have suggested that genetic factors may be account for approximately 50–65% of the risk of RA [2]. Human leukocyte antigen (HLA) alleles are well recognized to be implicated in the pathogenesis of RA [3]. However, family studies indicated that HLA alleles contribute to about 30% of genetic susceptibility and that non-HLA loci are also related to RA [2, 4].

Tumor necrosis factor alpha inducible protein 3 (TNFAIP3), a deubiquitinating protein, is reported to play a pivotal role in T cell activation and inflammatory signaling [5]. It can deregulate NF-κB-dependent gene expression via deubiquitinating specific NF-κB signaling molecules [6]. Genome-wide association studies (GWASs) have identified TNFAIP3 gene as a common genetic risk factor for RA [7, 8]. A host of studies [7, 9–21] investigated the associations between TNFAIP3 gene rs10499194, rs13207033 polymorphisms and RA susceptibility, but with conflicting findings. However, these studies were conflicting and inconclusive due to clinical heterogeneity, different ethnic populations, and small sample sizes. In order to precisely elucidate the genetic roles for TNFAIP3 gene polymorphisms (rs10499194, rs13207033) in the development of RA, we performed a comprehensive meta-analysis of identified studies to clarify the possible association between TNFAIP3 gene rs10499194, rs13207033 polymorphisms and RA risk.

## RESULTS

### Characteristics of the included studies

**Figure 1:**
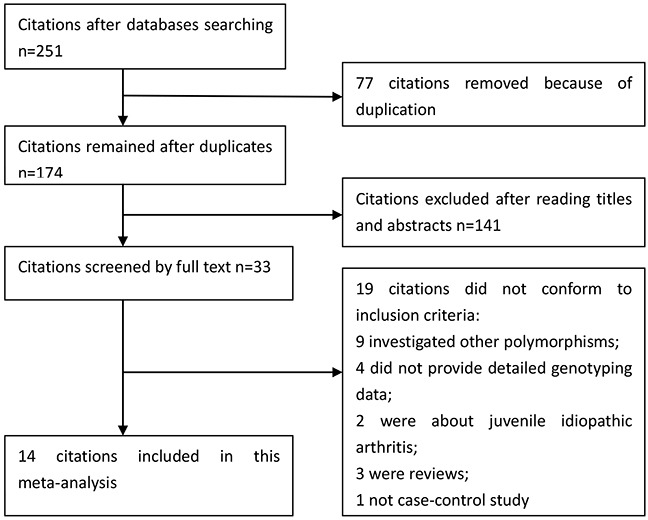
Selection for eligible publications included in this meta-analysis

We yielded a total of 251 citations after initial search. 77 citations were removed because of duplicates, and 141 citations were excluded after screening the titles and abstracts. 33 citations were selected for further full text review. 19 citations were excluded due to the following reasons: 9 investigated other polymorphisms; 4 citations did not provide detailed genotyping data; 2 were about juvenile idiopathic arthritis; 3 were reviews; and 1 was not case-control study. We finally identified 14 eligible citations [7, 9–21] including 17 studies (23,918 cases and 33,486 controls) in this meta-analysis. Selection for eligible studies included in this meta-analysis was presented in Figure 1. 6 studies with 11,166 cases and 11,231 controls examined rs13207033 polymorphism; 13 studies including 15,341 cases and 24,535 controls investigated rs10499194 polymorphism. The chaRacteristics Of Included studies are summarized in Table 1. The Newcastle-Ottawa Scale (NOS) scores of all included studies ranged from 5 to 7 stars, suggesting that these studies were of high methodological quality.

**Table 1:** Characteristics of included studies

Author and year	Country	Ethnicity	Case			Control			HWE	NOS
**rs10499194**			CC	CT	TT	CC	CT	TT		
Hegab_2016	Egypt	Caucasian	319	71	3	344	52	2	0.982	6
Zhang_2014	China	Asian	1156	12	4	1215	63	2	0.218	6
Hughes_2010	USA	African-American	374	164	18	558	213	20	0.951	7
Shimane_2010	Japan	Asian	2944	450	17	2049	243	7	0.943	6
Han_2009	Korea	Asian	1199	113	2	936	66	1	0.883	5
Coenen_2009	Dutch	Caucasian	790	499	79	972	614	97	0.998	7
Stark_2009	Slovakia	Caucasian	281	200	37	149	116	37	0.058	6
Perdigones_2009	Spain	Caucasian	305	275	46	306	287	70	0.824	6
Raychaudhuri_2008	Mixed	Caucasian	1353	1522	428	4636	5929	1895	0.993	7
Plenge_2007	USA	Caucasian	229	145	23	195	167	36	0.977	6
Plenge_2007	Sweden	Caucasian	560	280	35	519	276	37	0.968	6
Plenge_2007	North America	Caucasian	317	190	28	496	426	91	0.973	6
Plenge_2007	North America	Caucasian	491	327	55	673	604	136	0.977	6
**rs13207033**			GG	GA	AA	GG	GA	AA		
Zhang_2014	China	Asian	1040	232	8	1048	216	16	0.203	7
Han_2009	Korea	Asian	987	295	27	773	208	19	0.257	5
Maxwell_2012	UK	Caucasian	149	106	13	129	116	16	0.129	6
Orozco_2009	UK	Caucasian	2067	1403	224	1563	1237	229	0.464	7
Dieguez-Gonzalez_2009	Spain	Caucasian	825	684	142	800	676	143	0.991	6
Plant_2010	Mixed	Caucasian	1614	1148	202	2214	1555	273	0.999	6

HWE, Hardy–Weinberg equilibrium; NOS, Newcastle-Ottawa Scale

### Meta-analysis of TNFAIP3 gene rs10499194, rs13207033 polymorphisms

**Figure 2:**
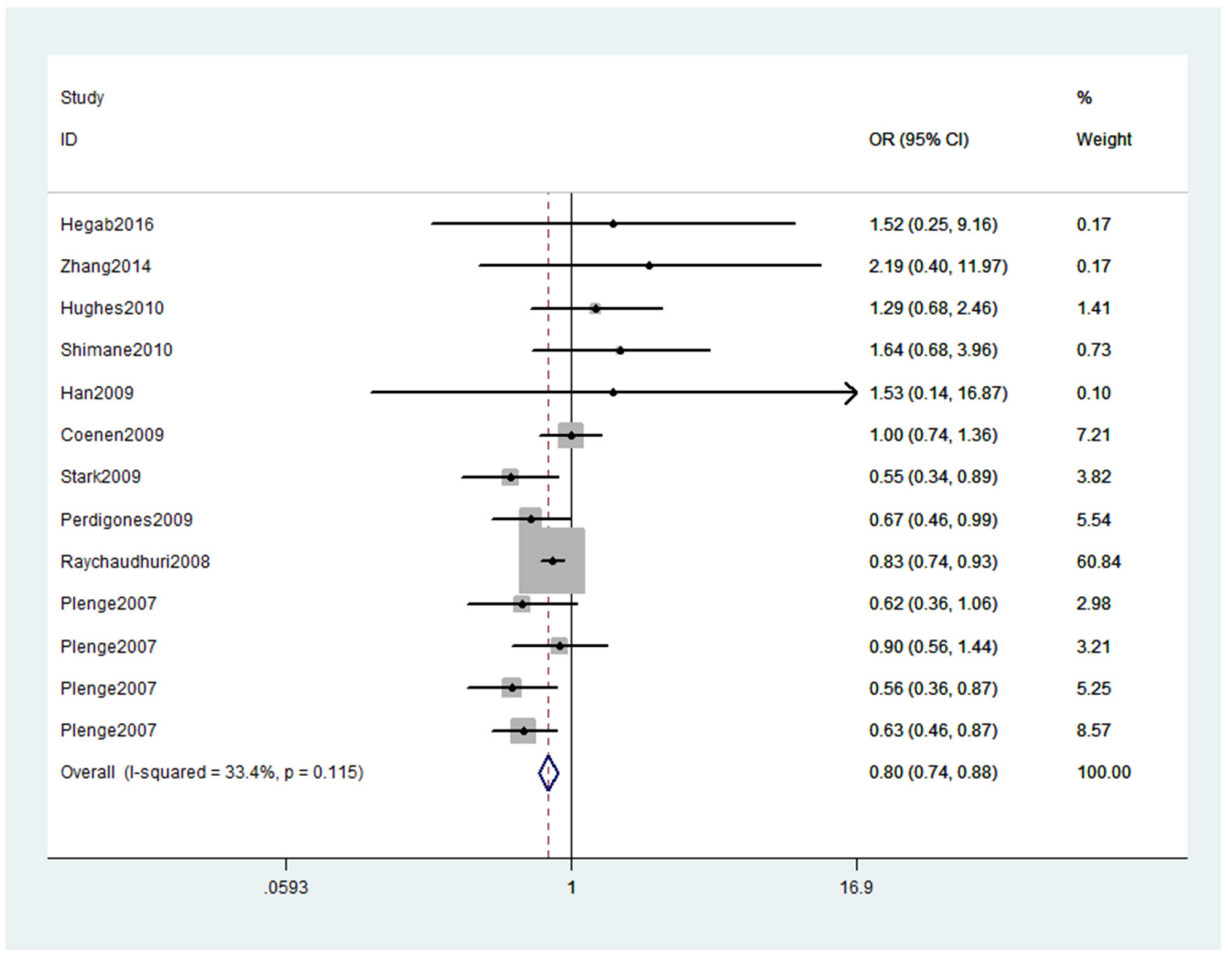
Forest plot shows odds ratio for the associations between rs10499194 polymorphism and RA risk (TT vs. CT+CC)

**Figure 3:**
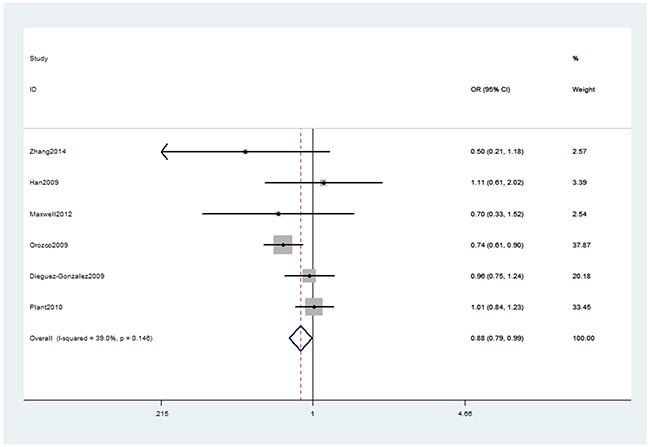
Forest plot shows odds ratio for the associations between rs13207033 polymorphism and RA risk (GA vs. GG)

**Figure 4:**
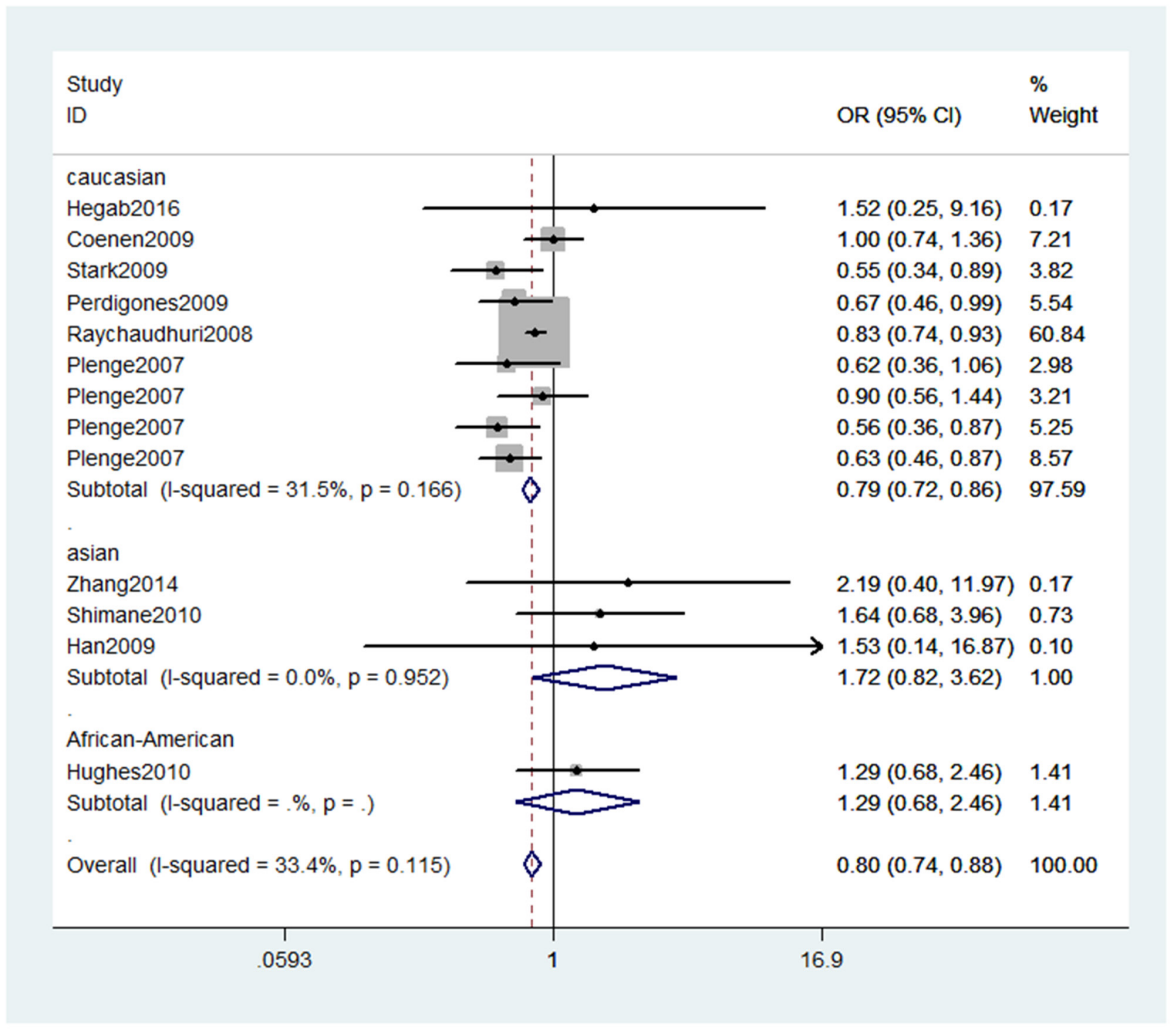
Stratification analyses of ethnicity between rs10499194 polymorphism and RA risk (TT vs. CT+CC)

In the overall analysis, we detected significant association (Table 2) between TNFAIP3 gene rs10499194, rs13207033 polymorphisms with decreased RA risk (rs10499194, TT vs. CT+CC: OR, 0.80; 95% CI, 0.74–0.88, *P* < 0.001, Figure 2; rs13207033, GA vs. GG: OR, 0.88; 95% CI, 0.79–0.99, *P* = 0.034, Figure 3). Stratification analyses were conducted according to ethnicity (Table 3). Our data indicated that rs10499194 polymorphism was also significantly associated with a decreased risk of RA among Caucasian populations (TT vs. CT+CC: OR, 0.79; 95% CI, 0.72–0.86, *P* < 0.001, Figure 4), but not among Asian and African-American populations. We also found rs13207033 polymorphism was weakly associated with a decreased risk of RA among Caucasian populations (GA vs. GG: OR, 0.88; 95% CI, 0.79–1.00, *P* = 0.044). All included studies conform to Hardy–Weinberg equilibrium (HWE), indicating control subjects were representative of the general population.

**Table 2:** Meta-analysis of association between TNFAIP3 rs10499194, rs13207033 polymorphisms and RA risk

Comparison	OR(95%CI)	*P*-value	*P* for heterogeneity	I^2^ (%)	Model
rs10499194					
T vs. C	0.91(0.81,1.02)	0.097	<0.001	85.0	Random
CT+TT vs. CC	0.90(0.78,1.03)	0.131	<0.001	84.9	Random
TT vs. CT+CC	**0.80(0.74,0.88)**	<0.001	0.115	33.4	Fixed
CT vs. CC	0.92(0.80,1.06)	0.239	<0.001	82.7	Random
TT vs. CC	**0.75(0.62,0.90)**	0.002	0.026	48.4	Random
rs13207033					
A vs. G	0.96(0.89,1.04)	0.349	0.023	61.8	Random
GA+AA vs. GG	0.96(0.87,1.06)	0.446	0.025	61.1	Random
AA vs. GA+GG	0.90(0.81,1.01)	0.080	0.305	16.9	Fixed
GA vs. GG	**0.88(0.79,0.99)**	0.034	0.146	39.0	Fixed
AA vs. GG	0.97(0.89,1.07)	0.556	0.062	52.4	Random

*Bold values are statistically significant (*P* < 0.05).

**Table 3:** Summary of the subgroup analyses in this meta-analysis

Comparison	Category	Category	Studies	OR (95% CI)	*P*-value
**rs10499194**					
T vs. C	Ethnicity	Asian	3	0.86(0.45,1.63)	0.638
		Caucasian	9	**0.86(0.78,0.94)**	0.001
		African–American	1	1.15(0.94,1.41)	0.173
CT+TT vs. CC	Ethnicity	Asian	3	0.81(0.40,1.65)	0.561
		Caucasian	9	**0.85(0.76,0.95)**	0.005
		African–American	1	1.17(0.92,1.47)	0.200
TT vs. CT+CC	Ethnicity	Asian	3	1.72(0.82,3.62)	0.153
		Caucasian	9	**0.79(0.72,0.86)**	<0.001
		African–American	1	1.29(0.68,2.46)	0.440
CT vs. CC	Ethnicity	Asian	3	0.75(0.35,1.61)	0.467
		Caucasian	9	**0.88(0.80,0.98)**	0.020
		African–American	1	1.15(0.90,1.46)	0.262
TT vs. CC	Ethnicity	Asian	3	1.75(0.83,3.68)	0.141
		Caucasian	9	**0.69(0.58,0.82)**	<0.001
		African–American	1	1.34(0.70,2.57)	0.374
**rs13207033**					
A vs. G	Ethnicity	Asian	2	1.05(0.92,1.19)	0.451
		Caucasian	4	0.93(0.85,1.03)	0.170
GA+AA vs. GG	Ethnicity	Asian	2	1.08(0.94,1.24)	0.296
		Caucasian	4	0.92(0.82,1.04)	0.174
AA vs. GA+GG	Ethnicity	Asian	2	0.83(0.52,1.34)	0.454
		Caucasian	4	0.91(0.81,1.02)	0.105
GA vs. GG	Ethnicity	Asian	2	0.85(0.53,1.37)	0.505
		Caucasian	4	**0.88(0.79,1.00)**	0.044
AA vs. GG	Ethnicity	Asian	2	1.10(0.95,1.27)	0.207
		Caucasian	4	0.93(0.84,1.03)	0.187

*Bold values are statistically significant (*P* < 0.05)

**Figure 5:**
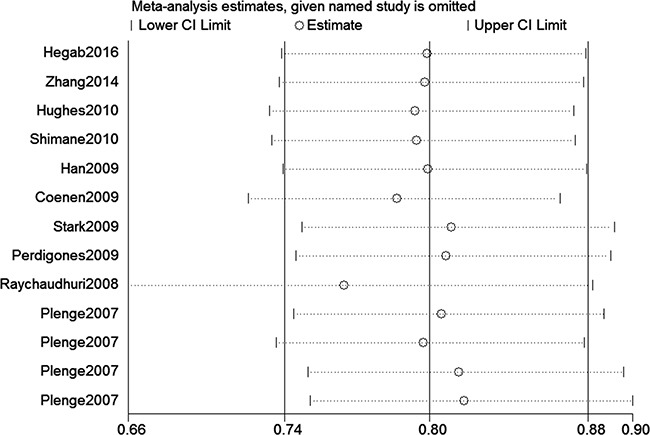
Sensitivity analyses between the rs10499194 polymorphism and RA risk (TT vs. CT+CC)

**Figure 6:**
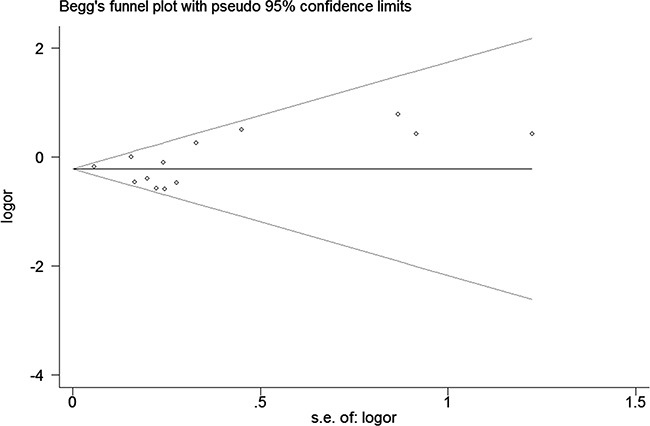
Begg's tests between rs10499194 polymorphism and RA risk (TT vs. CT+CC)

We assessed sensitivity by omitting each study once at a time in every genetic model for rs10499194 and rs13207033 polymorphisms. The pooled ORs for the effects of these two single nucleotide polymorphisms (SNPs) (rs10499194, TT vs. CT+CC: Figure 5) on the risk for RA risk indicated that our data were stable and trustworthy. Both Egger's and Begg's tests (rs10499194: TT vs. CT+CC, Figure 6) were used to evaluated the publication bias of this meta-analysis. Our data revealed that there was no obvious publication bias for rs10499194 and rs13207033 polymorphisms (data not shown).

## DISCUSSION

In this current meta-analysis, we found that TNFAIP3 gene rs10499194, rs13207033 polymorphisms were associated with the decreased risk of RA. Stratification analyses of ethnicity indicated that rs10499194 and rs13207033 polymorphisms decreased the risk of RA among Caucasians, but not among Asians.

Studies have demonstrated that NF-κB-dependent gene expression is significantly associated with the development of RA [21]. TNFAIP3 is an inhibitor of the NF-κB signaling pathway and it is indispensable in the development of RA [22]. Defects in TNFAIP3 expression are associated with development of RA [23]. Matmati et al. indicated that TNFAIP3 deficiency in myeloid cells triggers erosive polyarthritis resembling RA [24]. They revealed a pivotal and cell-specific function for TNFAIP3 in the pathogenesis of RA [24]. Recently, a number of [7, 9–21] studies explored the associations between TNFAIP3 gene polymorphisms (rs10499194, rs13207033) and RA risk. However, these studies obtained inconsistent results. The limitations of these studies including clinical heterogeneity, different ethnic populations, inadequate statistical power, and small sample sizes may contribute to the disaccord. Therefore, to obtain reliable conclusions, it is indispensable to conduct a comprehensive meta-analysis to demonstrate the associations between TNFAIP3 gene rs10499194, rs13207033 polymorphisms and RA susceptibility. Our data indicated that TNFAIP3 gene rs10499194 and rs13207033 polymorphisms decreased the risk of RA.

Previously, Lee et al. also performed a meta-analysis to investigate TNFAIP3 gene rs10499194 polymorphism with RA susceptibility [25]. They indicated that rs10499194 polymorphism was not associated with RA susceptibility [25]. Stratification analyses of ethnicity in their meta-analysis [25] suggested that rs10499194 polymorphism was weakly associated with an increased risk of RA among Asian populations, while no association was observed among Caucasian populations. However, our meta-analysis found that rs10499194 polymorphism was significantly associated with a decreased risk of RA among Caucasian populations, but not among Asian populations, indicating that diversity inheritance of different ethnicities. The reasons why the decreased risk of RA among Asians is not obvious are still unclear. The clinical heterogeneity, different ethnicities and small sample sizes may explain it. We think previous meta-analysis [25] conducted by Lee et al. had several limitations. First, Lee et al. did not include two studies [7, 16] which actually conformed to the inclusion criteria. Second, they identified a Spanish study [10] by Dieguez-Gonzalez et al., but we did not find the genotype numbers of rs10499194 polymorphism. Three, previous research indicated that searching one or two databases was insufficient for meta-analysis of case-control studies [26]. Lee et al. conducted a literature search only in the database of MEDLINE, but omitting Embase and other databases. Consequently, the reliability of their conclusions should be interpreted with caution. Due to these above limitations of previous meta-analysis, we re-conducted this meta-analysis. We believe our meta-analysis has some strength over previous meta-analysis for the following reasons. One, we identified 13 studies [7, 9, 11–13, 16, 18–21], including 15,341 cases and 24,535 controls with regard to rs10499194 polymorphism and the sample size of this meta-analysis was large enough. Two, sensitivity analysis indicated that our data about rs10499194 polymorphism were trustworthy and robust. Three, the power analysis indicated that our study had a power of 99.9% to detect the effect of rs10499194 polymorphism on RA susceptibility, assuming an OR of 0.80.

Regarding TNFAIP3 gene rs13207033 polymorphism, six studies explored the association between this SNP and RA risk. A study from UK [15] found rs13207033 polymorphism was significantly associated with a decreased risk of RA, but the remaining five studies [10, 11, 14, 17, 21] did not replicate the association. Therefore, we conducted the meta-analysis to demonstrate this SNP with RA risk. To our knowledge, this is the first meta-analysis to address the association between this SNP and RA risk. A weak association was obtained about rs13207033 polymorphism in our study. Stratification analyses by ethnicity also found a weak association between rs13207033 polymorphism and Caucasian RA risk.

Several potential limitations should be addressed in this meta-analysis. First, due to limited data, we could not perform further stratification analyses of other potential factors, such as anti-citrullinated peptide antibody (ACPA) and rheumatoid factor (RF). Second, our results were based on unadjusted estimates for confounding factors, which might have affected the final conclusions. Third, we could not assess potential gene-gene and gene-environment interactions because of the lack of relevant data. Fourth, the heterogeneity of this meta-analysis was high in some genetic models. Fifth, the conclusions about rs10499194 polymorphism among Asians should be interpreted with caution due to limited sample size.

In conclusion, this meta-analysis indicates that TNFAIP3 gene rs10499194, rs13207033 polymorphisms are associated with the decreased risk of RA, especially among Caucasians. Further studies are necessary to validate whether TNFAIP3 gene rs10499194, rs13207033 polymorphisms contribute to RA susceptibility in other ethnic groups.

## MATERIALS AND METHODS

### Literature search and criteria of inclusion

We systematically searched the PubMed, Embase, and China Knowledge Resource Integrated Database to identify studies through July 26, 2016. The following search terms were used: “tumor necrosis factor alpha inducible protein 3,” “TNFAIP3,” “A20,” “Rheumatoid Arthritis,” “RA,” “polymorphism,” “SNP” and “polymorphisms”. No restrictions were placed on the search. Additional initially omitted studies (such as reference lists of identified studies) have been identified by hand screening. The identified studies conformed to the following criteria: (1) studies that evaluated the association between RA risk and TNFAIP3 gene rs10499194 or rs13207033 polymorphism, (2) studied on human beings, (3) study provided sufficient data to calculate the odds ratios (ORs) and 95% confidence intervals (CIs), and *P* value, and (4) case-control study.

### Data extraction and quality assessment

Relevant information was carefully extracted from all eligible studies. The extracted information from all eligible studies including: name of first author, publication year, country of origin, ethnicity, and genotype numbers of cases and controls. Two reviewers independently performed the extraction of data and assessed the study quality based on the NOS [27]. All disagreements were discussed and resolved with consensus.

### Statistical analysis

All statistical analyses were performed using the Stata 11.0 software (StataCorp, College Station, TX, USA). ORs and 95%CIs were used to assess the strength of associations between TNFAIP3 gene rs10499194, rs13207033 polymorphisms and RA risk. Stratification analysis was carried out by ethnicity. *P* < 0.05 was considered statistically significant. When a Q test indicated *P* < 0.1 or I^2^ > 50% indicated heterogeneity across studies, a random-effect model was used. Otherwise, the fixed-effects model was applied [28]. Pooled ORs were calculated for allele model, dominant model, recessive model, homozygous model, and heterozygous model. We performed sensitivity analyses by omitting each study in turn to determine the effect on the test of heterogeneity and evaluated the stability of the overall results. We assessed the departure from the HWE in the controls using Pearson's χ2 test. Potential publication bias was assessed by Begger's and Egger's linear regression test [29]; *P* < 0.05 was considered to indicate statistically significant. The power of this meta-analysis was calculated with a significant value of 0.05 [30].

## Abbreviations

RA: rheumatoid arthritis
TNFAIP3: tumor necrosis factor alpha inducible protein 3
HLA: Human leukocyte antigen
CI: confidence interval
OR: odds ratio
NOS: Newcastle-Ottawa Scale
HWE: Hardy–Weinberg equilibrium
SNP: single nucleotide polymorphism
